# Advances in Laser Ablation Synthesized Silicon-Based Nanomaterials for the Prevention of Bacterial Infection

**DOI:** 10.3390/nano10081443

**Published:** 2020-07-24

**Authors:** Marina Martínez-Carmona, María Vallet-Regí

**Affiliations:** 1 Departamento de Oftalmología, Facultad de Medicina, Universidad de Murcia, 30100 Murcia, Spain; 2 Instituto Murciano de Investigación Biosanitaria Virgen de la Arrixaca (IMIB-Arrixaca), 30120 Murcia, Spain; 3 Department of Chemistry in Pharmaceutical Sciences, School of Pharmacy, Universidad Complutense de Madrid, Instituto de Investigación Sanitaria Hospital 12 de Octubre i+12, 28040 Madrid, Spain; 4 Centro de Investigación Biomédica en Red de Bioingeniería, Biomateriales y Nanomedicina (CIBER-BBN), Avenida Monforte de Lemos, 3-5, 28029 Madrid, Spain

**Keywords:** laser ablation, silicon, silica, nanoparticles, nanomaterials, bacteria, infection, biofilm

## Abstract

Nanomaterials have unique properties and characteristics derived from their shape and small size that are not present in bulk materials. If size and shape are decisive, the synthesis method used, which determines the above parameters, is equally important. Among the different nanomaterial’s synthesis methods, we can find chemical methods (microemulsion, sol-gel, hydrothermal treatments, etc.), physical methods (evaporation-condensation, laser treatment, etc.) and biosynthesis. Among all of them, the use of laser ablation that allows obtaining non-toxic nanomaterials (absence of foreign compounds) with a controlled 3D size, has emerged in recent years as a simple and versatile alternative for the synthesis of a wide variety of nanomaterials with numerous applications. This manuscript reviews the latest advances in the use of laser ablation for the synthesis of silicon-based nanomaterials, highlighting its usefulness in the prevention of bacterial infection.

## 1. Introduction

At a time in history when society looked “big” (bigger cars, bigger houses, bigger televisions, etc.) Richard Feynman turned back the tide and focused on the “small”. A new perspective that allowed us to rediscover everyday materials with new properties and applications. A representative example can be found in the evolution of carbon materials. In the past, only diamond, of great hardness, and graphite, easily delaminable and key element of pencils, were known. During the 50s, carbon fiber (formed by filaments of a few microns) emerged as a light and extremely resistant material that gained importance in aeronautics, the transport industry and high competition sports. Subsequently, the discovery of carbon nanotubes and more recently graphene, whose thermal and electrical properties greatly increase and even surpass that of conductive metals, revolutionized the field of carbon materials [1]. Silicon, with a chemistry similar to that of carbon and of great abundance in the earth, is another of the materials whose miniaturization has led to the appearance of new properties with application in numerous fields such as medicine, structural materials, agricultural field to the environmental bioremediation, solar cells, etc. [2,3,4]. Depending on the conditions and techniques used during the synthesis, it is possible to obtain two different types of silicon-based nanomaterials: (i) nanomaterials whose composition is identical to that of the original silicon (Si–Si), which from now on will be call silicon-nanomaterials (Si-NMs) or (ii) silicon oxide (silica, SiO_2_) nanomaterials that will be denoted as silica-nanomaterials (Silica-NMs). Undoubtedly, it is in medicine where the use of silicon-based nanomaterials has become most widespread. One of the main reasons is that silicon and silica nanomaterials have great mechanical resistance and thermal stability, which makes them ideal substrates for functionalization. This is a great advantage since it is precisely the possibility of anchoring a variety of elements on the surface of the nanomaterials, which allows modifying their properties depending on the necessary treatment. For example, silicon-based nanomaterials functionalized with antibodies [5,6], proteins [7,8], small molecules [9,10], etc., have been reported to increase treatment efficacy by selectively directing the nanoparticles (NPs) to the desired target (tumor cells, inflammation, infection, Alzheimer, etc.) [11,12,13,14,15,16,17,18]. What is more, the functionalization of these nanoparticles allows the creation of intelligent systems [18], that is, devices capable of responding to the presence of a stimulus from both, internal (pH, [19,20,21,22] enzymes [23,24]) or external (light [8,25,26], ultrasounds [27], magnetic field [26,28], etc.) origin.

Specifically in the treatment of bacterial infections, silicon-based nanomaterials have proven to be highly effective at several levels including early detection, targeting bacteria or biofilm and prevention on biofilm formation [17,29]. Its use as a drug delivery system allows to increase the effectiveness of the antibiotic and dose reduction, solving the problem of antibiotic resistance [30]. That is why its widespread use would be a great alternative to the synthesis of new antibiotics whose process requires a high investment of time and money [31]. Both, silicon and silica nanomaterials can be synthesized porously, increasing versatility since drugs, including poorly water-soluble ones, can be loaded into the pores and protected from the biological environment until the particle arrives to the target site. Besides, if necessary, larger pores can be obtained by modifying the surfactant type or by adding swelling agents, making it possible to house proteins and other bulky entities inside the pores.

On the other hand, silicon-based nanomaterials have been shown to be non-toxic to biological systems when prepared with the appropriate structural characteristics and applied in the correct doses [32]. Numerous studies have been carried out to investigate how size, shape, and other structural factors affect their biological activities such as: endocytosis through cell membranes, interferences within cell signaling, interaction with cell organelles, etc. [32,33]. Specifically, porosity turned out to be one of the key conditions that determine toxicity, efficiency of cellular uptake and immune response, and improving biocompatibility for porous forms [34].

Regarding the elimination from the body, biodegradation of silicon-based nanomaterials in vivo occurs mainly by dissolution in biological fluids. Its degradation takes place in the form of silicic acid, a compound naturally present in humans in small concentrations. Such degradation has been reported to take place faster in silicon nanoparticles [35] than in those composed of silicon oxide [36], and in both cases, porosity seems to accelerate the process [37,38] while functionalization can slow it down [39]. There are several studies that support the elimination of silicon-based materials through the renal and hepatic routes in the form of urine and feces that contain solid nanoparticles or degraded products, with renal excretion being the main route [35,36,39].

Furthermore, the widespread use of nanoparticles requires paying attention to the potential risks that their residues can have on the environment. Green algae, sensitive to a wide chemical range, is considered as an indicator of the industrial waste bioactivity. In one study, Oya N. et al. reported that unlike other oxide (ZnO, CuO, and TiO_2_) nanoparticles that turned out to be toxic, SiO_2_ nanoparticles not only did not inhibit the growth of algae but instead increased the growth rate [40].

Although the structure itself is not toxic, if not properly removed, residual surfactants (which are often cationic) and organic solvents (used during the synthesis process) can be detrimental to the integrity of cell membranes due to their lipophilic nature or the generation of free radicals, respectively [41].

Considering the increasing demand for silicon-based nanomaterials in biomedical applications, new chemical free synthesis alternatives methods are being developed. One of them is biogenic silica, that is, the formation of SiO_2_ by living organisms. Diatoms (unicellular and eukaryotic microalgae) [42] or siliceous sponges [43] are examples of organisms that developed silica-skeleton with an ordered three-dimensional structure on a micro- to nanoscale. However, the biogenesis process is accompanied by inorganic impurities and organic materials that must be removed before using the silica as a nanosystem. Said purification usually requires the use of chemical reagents and thermal processes, which, although to a lesser extent, move away from the desired eco-friendly synthesis [44]. Another alternative is laser ablation that enables nanoparticles production, in the absence of chemical compounds, by irradiation of larger materials [45]. Thus, different natural silicon sources (silicate rich garnet rocks [46], Rossella fibulata sponge [47], and sugarbeet bagasse [40]) have already been used to obtain silicon-based nanomaterials. This quick and easy to use technique, is governed by the parameters of the laser and the properties of the environment, is highly versatile and allows the control of the particle size [48]. This review article aims to provide an overview in laser ablation synthesized silicon-based nanomaterials, highlighting their advances in the prevention of bacterial infections.

## 2. Methodologies and Parameters that Affect the Production of Silicon-Based Nanomaterials

When a pulsed laser irradiates a material, its energy is partially absorbed by the material to a greater or lesser extent depending on its absorption characteristics. If the substrate is a metal, the transfer of energy causes the movement of the free electrons across the surface. However, in a non-metallic material bound electrons begin to vibrate, generating an increase in the temperature of the material. Depending on the amount of energy and the duration of the incident pulse, these temperature increase can cause different phenomena on the surface of the material, such as: fusion and vaporization, in addition, the ionization of the solid, liquid or vapor during laser irradiation can lead to the generation of plasma [49].

**Figure 1:**
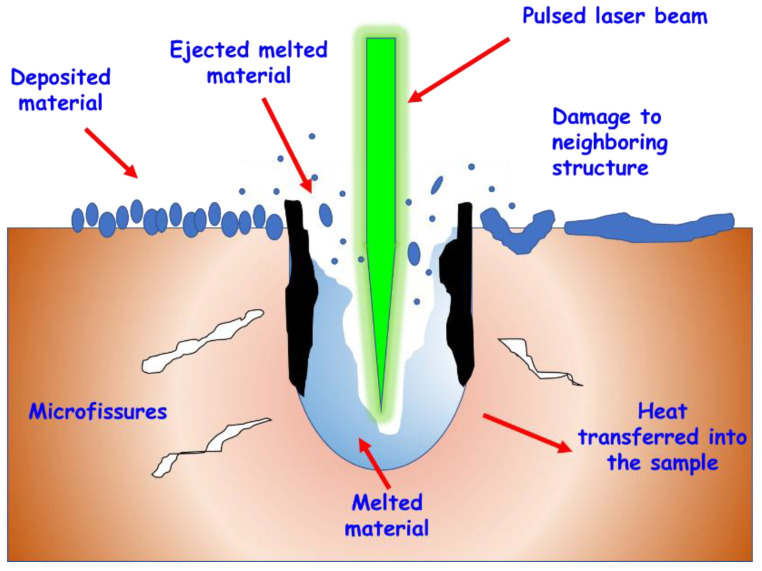
Ablation process observed during laser irradiation.

One of the phenomena that can be observed is the partial or total destruction of the material, an effect known as ablation. When the energy deposited is high enough, the vibration and the increase in temperature produce the electrons to detach from their bonds and break the atomic structure [50], managing to modify the material by an ionization process, that is, by creating plasma, leaving an empty space in the irradiation zone. Thus, laser ablation uses a high-energy pulsed laser to irradiate a substrate that, after absorbing the energy from the laser, is vaporized and subsequently forms a plasma. Laser ablation makes possible the micromachining of materials by creating cuts or engravings on the surface or, giving rise to nanomaterials from the material ablation cloud [51] (Figure 1).

**Figure 2:**
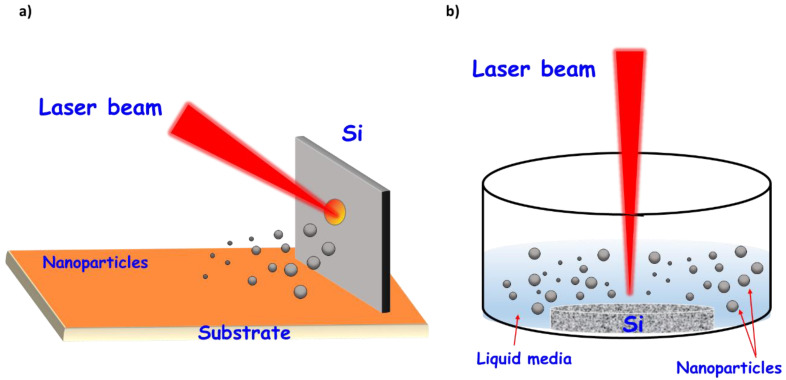
Ablation process performed in (**a**) “dry” and (**b**) “wet” conditions.

Nanoparticle formation begins with its nucleation during plasma cooling (at temperatures below the substrate melting point upon interaction with the environment), followed by nucleus growth and coalescence. Laser ablation can be performed both in “dry” (gaseous/vacuum ambient) or “wet” (liquid medium) conditions (Figure 2). When produced in a gaseous/vacuum environment, the formed nanoparticles can be gathered on a surface to create a nanostructured film or a powder-like deposit. Although there are modifications, the basic arrangement consists of a high intensity laser (10^8^−10^9^ W/cm^2^) that impacts on a silicon substrate located a few centimeters with an angle of about 45 degrees. To minimize energy losses and promote rapid cooling of the clusters, ultraviolet (UV) laser radiation is usually used through a plasma column [52].

On the other hand, when seeking colloidal nanoparticle suspensions, liquid ablation is the most widely used methodology. In this case, since no vacuum equipment is needed, the assembly is easier and consists of immersing the solid substrate in a liquid and performing the ablation using a laser vertically placed. The forming nanoclusters cool down in the liquid environment giving rise to a colloidal nanoparticle dispersion.

The laser-matter interaction has their origin in the energy transfer between the photons of the laser beam and the electrons of the material. Therefore, during the synthesis of nanomaterials by laser ablation, certain physical parameters of both the incident beam and the material to be processed must be considered.

In medical applications, especially for the infection treatment with drug delivery systems, smaller nanodevices that easily penetrate the bacterial wall are mainly used [53,54]. Although, bigger nanoparticles, whose mechanism of action is based on bacterial cell wall contact, are also reported [55]. Recently, it has been described that relatively large silicon-nanoparticles present great optical response in the visible and near IR ranges related with the excitation of optical magnetic and optical electric dipole resonances [56]. In any case, it is clear that regardless of the application, having control of the particle size obtained is essential. In the following sections we will review the latest published works paying special interest to those parameters that especially influence morphological characteristics.

### 2.1. Effects of the Ablation Method and the Ambient Media

As previously said, one of the main parameters to take into account when choosing the method is the type of product sought. To obtain a surface covered by small particles, vacuum/gas ablation is preferably the most used [57]. As showed by Gongalsky et al., the silicon-based nanomaterials can be subsequently removed from the substrate and dispersed in water by ultrasound application [58]. However, liquid ablation makes it possible to confine the plasma plume in a small region and directly obtain a dispersion of nanoparticles in the medium of interest [46,59].

During the generation of nanomaterials by laser ablation, the particles can react with the molecules in their environment, giving rise to oxides or other undesirable secondary products. Therefore, when designing the process, it is important to choose the medium carefully. “Dry” laser ablation can be performed both under vacuum and in the presence of an inert gas, however when it takes place under vacuum conditions it is difficult to control the growth of the nanoparticles, obtaining irregular distribution surfaces [60,61]. In contrast, when the synthesis occurs at moderate pressures, the established collisions between the atoms or molecules of the inert gas and the plasma allow to control the final size of the silicon-based nanomaterials preventing particles from coagulation [62,63,64]. On the other hand, the presence of an inert gas has not only been shown to affect size control but also material density. According to Kabashin et al. increasing the He pressure during ablation gave rise to a progressive evolution from dense Si/SiO*_x_* nanostructured films to highly porous ones (more than 90% of porosity) [65].

**Figure 3:**
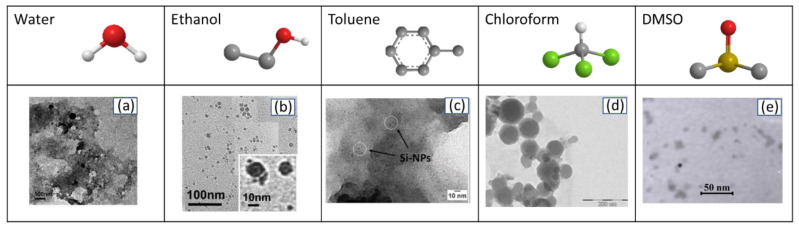
Summary of the silicon-based nanomaterials obtained by laser ablation of in different solvents: (**a**) water, (**b**) ethanol (Reproduced with permission from [71]. ACS Publications, 2009), (**c**) toluene (Reproduced with permission from [72]. Royal Society of Chemistry, 2012), (**d**) chloroform (Reproduced with permission from [73]. ACS Publications, 2011), and (**e**) dimethyl sulfoxide (DMSO) [71]).

Regarding the synthesis by “wet” laser methods, the solvent used has been shown to play a major role in the characteristics of the nanomaterials. Therefore, the effect of the solvent on the synthesis of silicon-based nanomaterials by laser ablation has been extensively studied (Figure 3). Distilled or deionized water is cheap, safe, does not absorb the light of the laser and have high heat capacity [66], reasons that make it the most widely used liquid medium for the synthesis of silicon-based nanomaterials by laser ablation [46,59,67,68,69]. When the synthesis takes place in an aqueous medium, the dissolved oxygen or the laser-induced decomposition products of the water can interact with the substrate giving rise to various species of oxide or hydroxide. Furthermore, these species, mainly the silanol groups on the surface, provide high Z potential values that favor obtaining stable nanoparticle dispersions [70].

Some organic solvents such as ethanol, chloroform, dimethyl sulfoxide (DMSO) and toluene have also been investigated (Figure 3). Yang et al. reported that under same conditions Si-NPs prepared in ethanol were ultrafine, single crystalline and exhibited superior dispersibility to those acquired in water [72]. SiO_2_ porous nanoparticles with an average radius of *r* ≈ 6.5 nm were also produced in ethanol [73]. Ultrafine Si-NPs were also synthesized in toluene [74]. However, besides Si-NPs, also SiC-NPs with moissanite 3C phase were observed, being both surrounded by a black carbon solid film attributed to toluene byproduct produced during the ablation process. Abderrafi et al. used chloroform as a medium with the purpose of taking advantage of the interactions with halogen-substituted hydrocarbons to limit the growth of silicon nanostructures, obtaining nanoparticles of about 50 nm in diameter [75]. When laser ablation is performed in DMSO spherical silicon nanocrystals with size range of 2–5 nm are achieved [71]. The ultrafine size of the nanoparticles was attributed to the DMSO oxide formations on the particle surfaces. It has also been proposed that the high dipole moment of organic solvents can generate a stronger electric double layer that enhances repulsive force between nanocrystals favoring the formation of smaller entities [76].

### 2.2. Effects of Laser Processing Parameters

Laser parameters such as wavelength, pulse intensity and pulse duration play a crucial role in size and other nanoparticles properties.

Intartaglia reported that in solution IR irradiation produced silicon-nanoparticles with a mean diameter of 40 nm, regardless the ablation time, while using UV laser resulted in 9 nm for short ablation time and 3 nm for longer periods. The authors attributed this to a photo-fragmentation process that greatly differ for both wavelengths. In the case of the UV ablation process the first synthesized 9 nm Si nanoparticles diffuse in the solvent and absorb the UV laser pulses producing ultra-small nanoparticles. However, during IR ablation there is no interaction between the laser and the produced nanoparticles giving rise to bigger nanoparticles [69]. In a different study performed by Rawat et al. the ablation time showed low influence on the size of the particles but a great effect on their composition. Thus the irradiation of garnet substrate for 20 min gave rise to a mixture of Si and SiO_2_ nanoparticles in proportion (30:70) while increasing the ablation time above 30 min practically entails the elimination of the Si particles that oxidize to SiO_2_ because of the high temperature originated in the vicinity [46].

Pulse duration determines the speed of interaction between the pulse energy and the material. Pulse duration can range from nano- to femto-seconds strongly influencing the type of physical interaction that take place since the relaxation time of the material is usually in the order of the pico- or nano-seconds [77]. Based on this it is possible to distinguish two different regimes of laser-matter interaction: (i) pico- and nano-seconds, being the duration of the pulse greater than the material relaxation time. In this case part of the energy can act in the vicinity in the form of heat, affecting the irradiated area but also the surroundings and (ii) femtoseconds, being the duration pulse shorter than the relaxation time. The duration is short, so the heat has no time to be transmitted, minimizing the damage to the irradiation area. The repetition frequency is defined as the number of pulses emitted per second [78,79].

It has been described that laser ablation in liquids is accompanied with cavitation bubble appearance [80]. Bubbles that, depending on their lifetime and diameter, can interact with the nanoparticles in formation, modifying their size. Three different scenarios are possible: the distance between consecutive laser pulses is higher than cavitation bubble size, laser pulses come one after another immediately (0% overlapping), and 50% pulse overlapping. Krivonosov tested the effect of these three situations on the synthesis of silicon nanoparticles by fixing pulse duration in 50 ns and fluence in 10 J/cm^2^ [67]. Results showed that for distances between pulses below to 50 µm, laser radiation is partially blocked by growing cavitation bubble, providing an increase in heat and agglomeration in the bubble. Contrary, because of partial loss of laser power, the heating experimented by the target is decreased, reducing the concentration of silicon nanoparticles in the solution. The optimal distance between laser pulses turned out to be 350 μm for the generation of uniform nanoparticles at the highest concentration.

On the other hand, if the target is larger nanoparticles (100–400 nm), the application of femtosecond laser ablation in air appeared to be the most effective pulse duration [81,82,83]. Popovic et al. showed that introducing the continuous wave laser in the ablation process leaded to an increase in crystallinity and in the photoluminescence of the nanomaterials [59].

As previously discussed, selecting the media and fixing the laser processing parameters allows control of particle size to some extent. However, coagulation is a complex process which usually involves size variability, therefore post-processing separation methods are commonly used. Some of them are quite simple, for instance, in “dry ablation”, achieving size selection varying the distance of the deposit from the target [84]. This method is based on the fact that nanoparticles of different sizes are ejected at different speeds from the subtract and therefore, smaller particles, that fly faster, are deposited at greater distances [85]. There are other more sophisticated such as the one reported by El-Shall, who applied an external gradient temperature to control the plasma plume evolution. Additionally, especially for “wet synthesis” traditional separation techniques such as filtration, centrifugation, ultrasonic treatment, electrophoresis, etc. can be used [72,75].

## 3. Antibacterial Effect of Silicon-Based Nanomaterials

**Figure 4:**
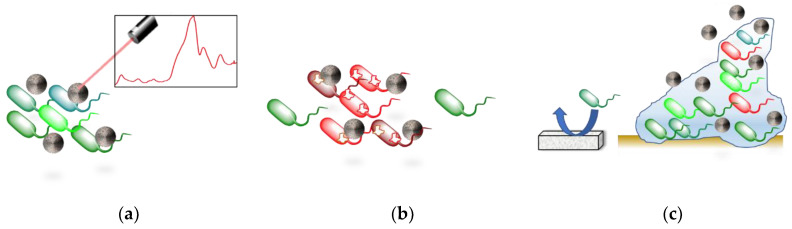
Summary of the silicon-based nanomaterials used for the treatment of bacterial infection depending on the stage in which they operate. (**a**) Bacteria Detection [86,87,88,89,103]. (**b**) Effect on Planktonic Bacteria [86,90,91,92,93,94,95,96,97,100,103]. (**c**) Effect on Biofilm [90,104,105,106,107].

Over time, bacteria are becoming more resistant to the use of antibiotics, which forces the investigation of new treatments for fighting bacterial infections. One of the alternatives that is gaining importance to solve this problem is the use of nanomaterials for drug delivery, reducing the dose and increasing the effectiveness of antibiotics. Recently, many studies have reported the value of silicon-based nanomaterial treatments at different stages of bacterial infection, from its early detection to its destruction once the biofilm has been formed (Figure 4).

Precise and rapid diagnosis of bacterial infections is crucial in order to prevent future complications and minimize the amount of antibiotics needed. A variety of diagnostic silicon-based nanomaterials with diversity in selectivity, sensitivity and efficacy (depending on the imaging agent and targeting ligand incorporated) have been published. For instance, Zhai et al. synthesized vancomycin modified fluorescent silicon nanoparticles (SiNPs-Van) able to selectively detect Gram-positive bacteria both, in vitro and in vivo [86]. The probe, in addition to following infection for 8 days, showed twice as much antibiotic efficacy as that of free vancomycin. A similar device but using mesoporous silica nanoparticles as a substrate was reported by Qi et al. [87]. In a different study, the functionalization of the surface of acridine orange-labeled silica nanoparticles with amino groups shown to be suitable for the detection of Gram-positive *Staphylococcus aureus* bacteria [88]. Wang and Kang designed a bioprobe silica nanoparticle doped with a fluorescent ruthenium complex and functionalized with single-stranded DNA aptamers for the detection of *Salmonella typhimurium* Gram-negative bacteria [89].

Focusing not on detection but on infection treatment, there are numerous publications in the literature on the use of silicon-based nanomaterials to combat bacteria. Most of them owe their antibacterial action to the release of antibiotics such as: levofloxacin [90,91,92], tetracycline [93], rifampicin [94], etc. However, it can also be due to the action of: (i) tandem peptides, one as membrane-targeting element and one as a toxic peptide cargo [95]; (ii) photodynamic therapy, which combines the nanoparticles with nontoxic dyes that when irradiated with light produce reactive oxygen species killing bacterial cells [96,97]; (iii) the release of nitric oxide. The reductive capacity of radical NO to produce lipid peroxidation and bacteria’s wall disruption has been known since 1990 [98], however, limitations regarding low storage capacity and uncontrollable release rates set aside their use for a long time. Its incorporation in silicon [99] and silica [100] nanoparticles overcome these problems recovering its interest as a bactericide; and iv) the combination with some metals such as copper, silver, zinc, etc. that have well known antibacterial properties [101,102].

In some cases, nanosystems combine several of the effects previously described, for example, Tang et al. reported a multifunctional silicon nanoagent able to detect ant effectively deal infections caused by diverse Gram-negative and Gram-positive bacteria [103]. The nanoagents were functionalized with a glucose polymer (poly[4-O-(α-D-glucopyranosyl)-D-glucopyranose]), serving as targeted ligand for bacteria, and loaded with chlorin e6 acting both: as an imaging agent and as a photodynamic therapy agent.

Finally, regarding the last phase of infection, the biofilm formation, silicon-based nanomaterials have also shown a very relevant role. Jeong et al. created a silicon nanowire array platform to study the effect of nanoscale topography in bacterial movement and attachment. Results obtained by single-cell imaging evidenced that *Shewanella oneidensis* MR-1 recognized and showed preferential attachment to the nanowires [104]. Conversely, Cousins et al. observed that the deposition of silica nanoparticles on a polystyrene surface reduced the attachment of *Candida albicans* [105]. More experiments would be needed to draw any conclusions about whether these differences are due to the type of bacteria, the composition or the morphology of the deposited nanomaterial or a combination of all of them. Once the biofilm is formed, the surface functionalization of silicon-based nanomaterials with metal nanoparticles [106], bacteriophages [107], or lectins [90] probed to be effective for biofilm disruption.

## 4. Application of Silicon-Based Nanomaterials Synthesized by Laser Ablation for Combating Bacterial Infection

In the previous section, many applications were described that silicon-based nanomaterials present to combat bacterial infection. Silicon-based nanomaterials synthesized by laser ablation are structurally analogous and therefore possessors of all of them, with the additional advantage of not using chemical reagents during formation, reducing the potential toxicity in the body. Surprisingly, its use so far is limited. Among the possible stages for the treatment of bacterial infection (detection, action on planktonic bacteria, and action on the biofilm) that can be intervened, the use of silicon-based nanomaterials created by laser ablation, has preferably focused on the creation of anti-adherent surfaces to prevent the biofilm formation. Reason why the present manuscript focuses on describing its usefulness in this stage.

### 4.1. Silicon-Based Nanomaterials Synthesized by Laser Ablation for Preventing Bacterial Infection

**Figure 5:**
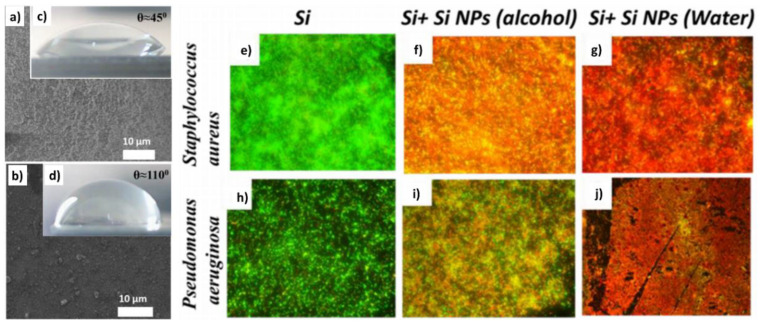
Top-view SEM images of coatings made of silicon nanoparticles (NPs) prepared in (**a**) water and (**b**) alcohol. Contact angles of wetting for the coatings prepared in (**c**) water and (**d**) alcohol. Optical images of assays of live (green) and dead (red) cells from bacteria biofilms on: bare silicon wafer (**e**,**h**), on silicon NP (prepared in isopropyl alcohol) coating on the silicon wafer (**f**,**i**) and on silicon NP (prepared in water) coating on the silicon wafer (**g**,**j**) (Reproduced with permission from [108]. IOP Publishing, 2018).

Smirnov et al. prepared silicon nanoparticles by means of the laser ablation of a solid silicon target in water (Figure 5a) and isopropyl alcohol media (Figure 5b) [108]. These nanoparticles formed a uniform surface coating onto a silicon wafer and their hydrophobicity and antifouling ability were tested. For studying the hydrophobicity, a drop of water was added on the top of the materials and the contact angle was measured. Silicon nanoparticles obtained in alcohol probed to have a greater hydrophobicity (Figure 5c,d).

Regarding the antibacterial effect of the nanocoating, both of them presented stronger inhibitory effect on the *S. aureus* and *P. aeruginosa* biofilm formation compared to the non-ablated silicon sample. The authors attribute this antimicrobial effect to the generation of reactive oxygen species formed on the surface of Si nanoparticles during their production. Lately, an study in terms of productivity for nanosecond-laser plasma-mediated ablation regimes of these nanoparticles in water was performed [109].

**Figure 6:**
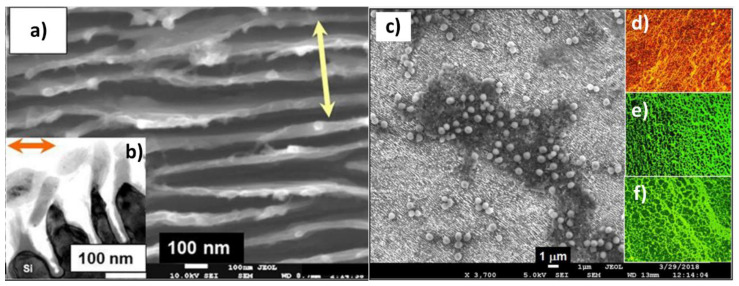
(**a**) High-magnification top-view SEM and (**b**) side-view cross-sectional TEM (inset) images of the surface nanoripples. (**c**) Oblique-view (20°) SEM image of the nanosharp Si pattern with the inactivated *Staphylococcus aureus* bacterial culture (light beads). Right insets (**d**–**f**): Microscopic images of stained bacteria on the nanopatterned (dead “red” bacteria, top) and smooth (live “green” bacteria, middle) Si and the control silica glass slide (live “green” bacteria, bottom) after 24 h of incubation. The frame sizes are 60 × 90 μm. (Reproduced with permission from [112]. ACS Publications, 2018).

Sometimes the laser ablation of the substrate does not lead to the formation of nanomaterials but to certain periodically aligned nanostructures that are called LIPSS (Laser-Induced Periodic Surface Structures). Controversy exists over the process by which these structures are formed. Some authors attribute them to a process of interference between the incident electromagnetic wave and that reflected and scattered by the material that gives rise to a pattern that affects only certain parts of the surface [110]. Others propose that it is the result of self-organization of the material surface during relaxation after application of the pulses [111]. Kudryashov et al. used picosecond IR-laser pulses to irradiate a Si wafer surface in liquid CS_2_ to produce Si ripples nanosheet arrays via nanoplasmonic ablative self-organization (Figure 6a,b) [112]. The antifouling capacity of the material was tested by culturing the nanosheet with *Staphylococcus aureus* bacteria for 18 h (Figure 6c). “Live/Dead Biofilm Viability Kit” was used to study the viability of bacteria, showing the absence of the biofilm and the death of almost all the bacteria (Figure 6d). Contrary, the appearance of a biofilm was observed for both controls: smooth Si wafer (Figure 6e) and silica glass slide (Figure 6f).

In addition to the usefulness of pure silicon-based nanomaterials in preventing bacterial infection, some articles have reported that their combination with other materials or small molecules (such as antibiotics) that have bactericidal properties improves its action against microorganisms.

**Figure 7:**
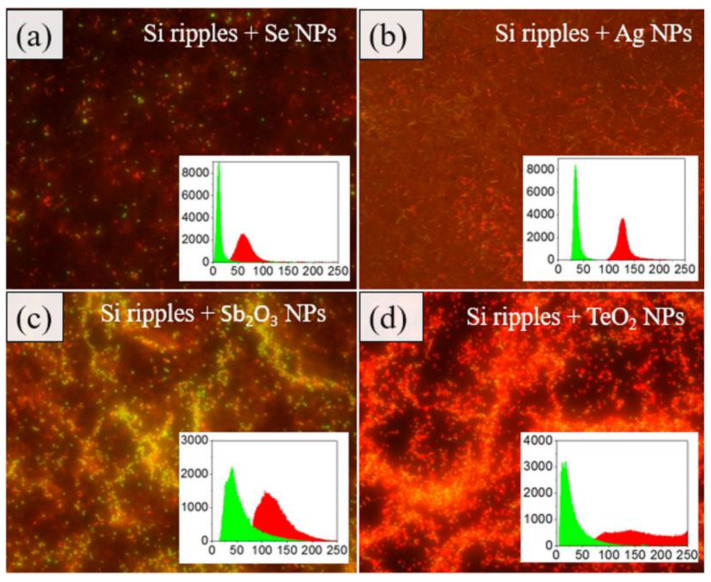
Fluorescent microscope photographs of dye-stained *S. aureus* bacteria cultured on Si ripples covered with (**a**) Se NPs, (**b**) Ag NPs, (**c**) Sb_2_O_3_ NPs, and (**d**) TeO_2_ NPs. (Reproduced with permission from [113]. IOP Publishing, 2020).

For instance, it was observed that the coating of Si ripples nanosheets with Se, TeO_2_, Sb_2_O_3_, and Ag NPs nanoparticles, capable of damaging the bacterial DNA by the production of ROS, increased the antibacterial properties of the surfaces. Being the sample that combined Si nanoripples and TeO_2_ the most effective for biofilm prevention (Figure 7) [113].

**Figure 8:**
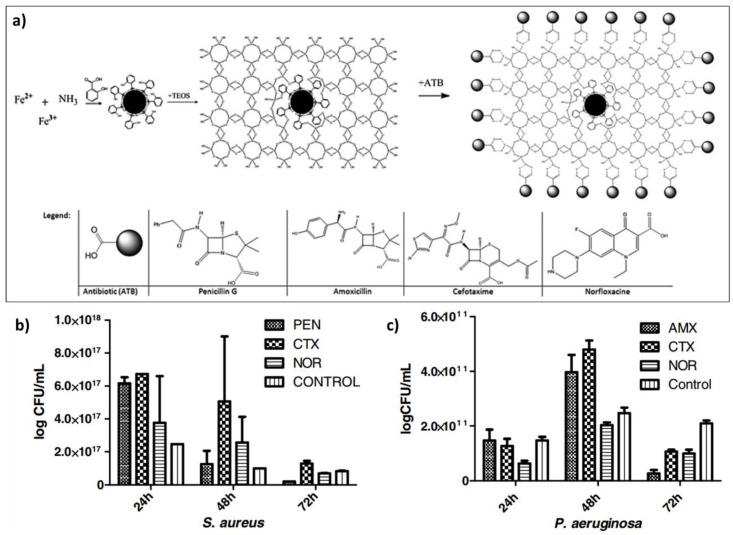
(**a**) Synthesis of Fe_3_O_4_/SA/SiO_2_/ATB nanoparticles. The gray sphere represents the antibiotic (ATB) molecule. The comparative representation of the viable cell counts recovered from the (**b**) *S. aureus* biofilms and (**c**) *P. aeruginosa* developed on MAPLE-deposited thin films at 400 mJ cm^−2^. (Reproduced with permission from [116]. IOP Publishing, 2012).

The addition of antibiotics to the silicon-based nanostructures is another strategy to increase the antibacterial effect. For most of the materials the ablation temperature is lower than the decomposition temperature, however, for some polymers, biomolecules, proteins, antibiotics, etc. this standard is not followed. These materials are very sensitive to temperature and degrade easily. In order to achieve a fine coating of these organic materials, a modification called matrix-assisted pulsed laser evaporation (MAPLE) is used [114]. In MAPLE, the sensitive compound is dissolved or dispersed in an inert solvent and then frozen to form a solid substrate. During laser ablation the volatile solvent absorbs most of the laser energy whereas the intact molecule of interest acquires enough kinetic energy to be transported and form a uniform film on the desired surface [115]. This variation was used by Mihaiescu et al. to create magnetite/salicylic acid/silica shell/antibiotics thin films [116]. The preparation of Fe_3_O_4_/SA/SiO_2_ nanoparticles as well as the studied antibiotics are schematically depicted in Figure 8. The authors reported that despite the differences found for both types of bacteria (*S. aureus* and *P. aeruginosa*), the thin films exhibited an inhibitory effect for the biofilm formation.

### 4.2. Silicon-Based Nanomaterials Synthesized by Laser Ablation for Bacterial Detection

**Figure 9:**
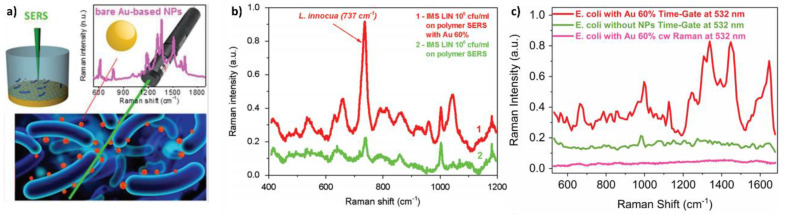
(**a**) Schematic description of the research carried out. (**b**) Normalized surface-enhanced Raman scattering (SERS) spectra of immunomagnetic separation (IMS) bead captured *L. innocua* (IMS LIN) detected using a polymer-based SERS substrate (green) and a combination of the substrate and Au-60% NPs (red) using 785 nm CW excitation. (**c**) SERS spectra of *E. coli* W3110 measured with time-gated Raman using 532 nm wavelength picosecond pulsed excitation with the use of Au-60% NPs as Raman probes (red) and without them (green). For comparison the Raman spectra without time gating (pink) is shown. (Reproduced with permission from [117]. John Wiley & Sons, 2018).

Although the application of these nanomaterials to prevent bacterial infection is the majority, an article was recently published highlighting their usefulness as a probe. Kögler et al. combined the ability of gold nanoparticles to enhance Raman scattering (surface-enhanced Raman scattering, SERS) with the silicon biocompatibility to create mobile SERS probes for bacteria detection (Figure 9a) [117]. Three types of gold nanoparticles with increasing concentrations of silicon (0%, 40%, and 70%) were synthesized. As the amount of silicon increased, the size of the particles and the capacity to serve as SERS probes (assessed by studying the enhancement of Rhodamine 6G as a model molecule) decreased. Gold 60% nanoparticles were selected as optimal and used to test their applicability as biosensors against two different bacteria species: *L. innocua* and *E. coli*. As seen in Figure 9b Raman spectra of *L. innocua* using only the patterned SERS substrate or combined with Au-60% NPs gave rise to similar peaks, however, the intensity of the signal for the combined detection was 4.4-times higher. When the experiment was carried out in the presence of *E. coli* (Figure 9c), the autofluorescence of the bacteria was so high that it prevented their detection (pink line). However, the use of time-gating solved the autofluorescence problem and allowed to register a spectrum that in the presence of nanoparticles was more than one order of magnitude greater than only with the substrate and which revealed a variety of novel Raman lines.

## 5. Conclusions and Future Perspectives

The mechanisms of laser ablation for obtaining silicon-based nanomaterials is well documented, however, there is little information about their application for the treatment of bacterial infection. This implies that much remains to be discovered on this topic and therefore it is an interesting field to investigate. Most of the publications related to the use of Silicon-based nanomaterials synthesized by laser ablation for the treatment of infection focus on the creation of non-stick surfaces. It would be of great scientific value to study the antibacterial effect that these nanomaterials have in the different stages of infection and to see if the same material can have a long-lasting efficient action. In the same way, a comparative study between silica and silicon nanomaterials would provide great information on the similarities and differences of both materials with respect to antibacterial activity, allowing the most suitable material to be chosen in each case. Furthermore, since silicon-based nanomaterials produced by laser ablation are of the same nature as silicon-derived nanomaterials synthesized by chemical and biogenic methods, it would be of great interest to synthesize equivalent materials by these three routes and compare their effects on various bacterial strains. These studies would shed light on whether the synthesis method itself has any effect on the antibacterial activity of nanomaterials.
